# Tumor-Related Exosomes Contribute to Tumor-Promoting Microenvironment: An Immunological Perspective

**DOI:** 10.1155/2017/1073947

**Published:** 2017-05-31

**Authors:** Wuzhen Chen, Jingxin Jiang, Wenjie Xia, Jian Huang

**Affiliations:** ^1^Department of Surgical Oncology, Second Affiliated Hospital, Zhejiang University School of Medicine, Hangzhou, China; ^2^Cancer Institute (Key Laboratory of Cancer Prevention & Intervention, National Ministry of Education, Provincial Key Laboratory of Molecular Biology in Medical Sciences), Zhejiang University School of Medicine, Hangzhou, China

## Abstract

Exosomes are a kind of cell-released membrane-form structures which contain proteins, lipids, and nucleic acids. These vesicular organelles play a key role in intercellular communication. Numerous experiments demonstrated that tumor-related exosomes (TEXs) can induce immune surveillance in the microenvironment in vivo and in vitro. They can interfere with the maturation of DC cells, impair NK cell activation, induce myeloid-derived suppressor cells, and educate macrophages into protumor phenotype. They can also selectively induce effector T cell apoptosis via Fas/FasL interaction and enhance regulatory T cell proliferation and function by releasing TGF-*β*. In this review, we focus on the TEX-induced immunosuppression and microenvironment change. Based on the truth that TEXs play crucial roles in suppressing the immune system, studies on modification of exosomes as immunotherapy strategies will also be discussed.

## 1. Introduction

Traditionally, we always consider neoplasm as a disease driven by the alteration of the cellular genome, with overexpression of oncogenes, or deficiency of tumor suppressor genes [1]. After decades of hypothesis and proof, now, it is widely accepted that tumor microenvironment and their interactions with the host immune system are important in tumor genesis and progression [2–4]. Although the host immune system mostly works well at eliminating tumor cells, defenses are sometimes blunted by the activation of suppressive pathways which degrade immune restraints on tumor spreading [5, 6]. Exosomes, a branch of extracellular vesicles, were termed by Trams in 1981 for exfoliated membrane vesicles with 5′-nucleotidase activity. Generally, exosomes are described as 30 to 100 nm size exfoliated vesicles originated from the endosome organelles, which are different from macrovesicles (>100 nm) in size [7]. According to present studies, exosomes are accumulated in the multivesicular bodies and released to the extracellular environment through fusion of multivesicular bodies with the plasma membrane. Exosomes can be released by all types of cells including cancer cells, fibroblast cells, immune cells, and mesenchymal cells. The contents of exosomes, including a cargo of different genes, lipids, proteins, and miRNAs, are mostly defined by their parental cells. Being able to stably transfer their contents to distant sites, exosomes have been proved to be an effective mode of cross talk among cells far apart and are involved in multiple physiological and pathological processes.

Cancer cells have built such a subtle and sophisticated intercellular communication with the host environment and facilitate tumor progression through secreting exosomes, including the process of tumorigenesis, tumor growth, invasion, and metastasis. Tumor-derived exosomes (TEXs) were initially discovered from peripheral circulation and malignant effusions in ovarian cancer patients [8–10]. After then, exosomes were found in many other malignancies such as breast cancer and colon cancer [1, 11, 12]. TEXs emerged as a new pattern of intercellular communication and play a crucial role in the tumor microenvironment. Previous studies have demonstrated that TEXs play an essential role in tumor angiogenesis, matrix remodeling, tumor migration, and metastasis [13]. Stress, microenvironment hypoxia, and activation of wild-type p53 would lead to more releases of tumor-related exosomes [14, 15]. Apart from tumor cells, exosomes can be released by a variety of activated immune cells, including dendritic cells (DCs), macrophages, B cells, T cells, and natural killer (NK) cells [16–18]. These organelles make available for strategic function in intercellular communication and regulation of immune responses. The immunosuppression role of TEXs have been widely indicated and furtherly result in the tumor progression outcome. TEXs can participate in multiple immune mechanisms, such as mast cell degranulation, germinal center reaction, and cell apoptosis, with a consequent downstream blockade in the natural antitumor immune responses [13]. Western blots of TEXs isolated from tumor cell supernatants and exosome fractions obtained from cancer patients' plasma confirm the expression of various immunosuppressive molecules, including death receptor ligands such as FasL and TRAIL, checkpoint receptor ligands such as PD-L1, and inhibitory cytokines such as IL-10 and TGF-*β*, as well as prostaglandin E2 (PGE2) and ectoenzymes engaged in the adenosine pathway (CD39 and D73) [19]. A large amount of TEX-derived noncoding RNAs are also regarded as immunosuppressive agents. Obviously, it is a complex network involving TEXs and the host immune system, together with the mechanisms of exosome, which mediate phenotypical and functional defects. In the following paragraphs, this review will discuss the moderating role of TEXs on multiple immune cells such as DCs, NKs, macrophages, effector T cells, regulatory T/B cells, and myeloid-derived suppressor cells (MDSCs). For practical applications, the new immunotherapy strategy based on DC-derived exosomes and bioengineering of exosomes will also be illustrated in this work.

## 2. Dendritic Cells

**Figure 1:**
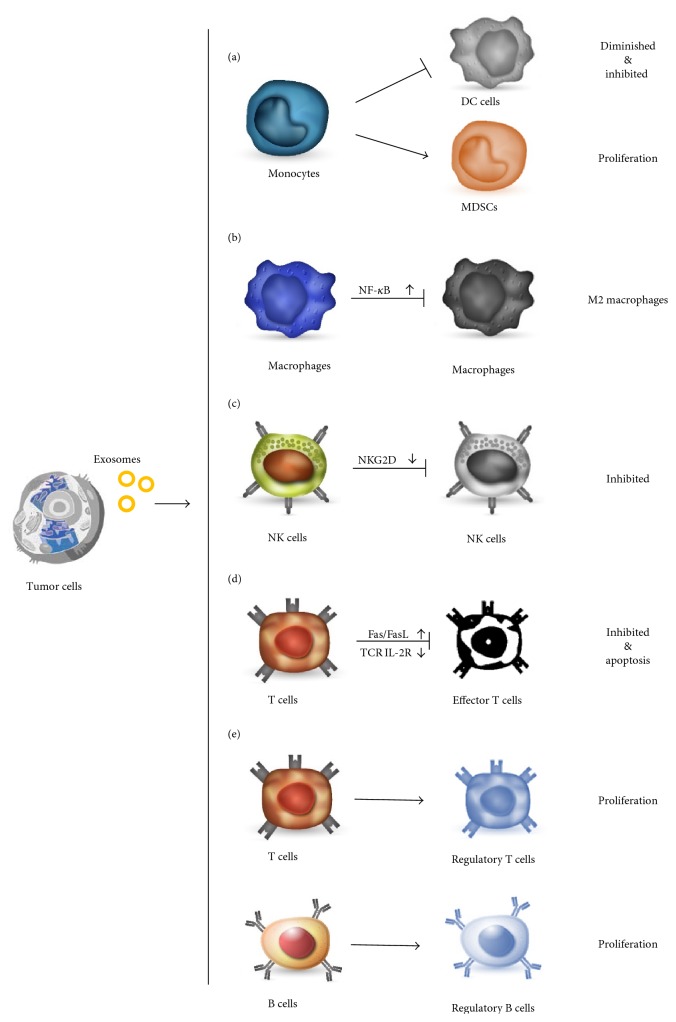
Tumor-released exosomes could mediate immune suppression. (a) TEXs could induce peripheral monocyte differentiating into MDSCs instead of DCs and inhibit DCs' bioactivity. (b) TEXs stimulate NF-*κ*B signals in macrophages and induce them into the M2 cytokine profile. (c) TEXs downregulate NKG2D and inhibit the cytolytic activity in NK cells. (d) TEXs inactivate effector T cells by interfering with TCR- and IL-2R-mediated signaling and induce effector T cell apoptosis via Fas/FasL interaction. (e) TEXs contribute to regulatory T cells proliferation via TGF-*β* and IL-10 and transform normal B cells into regulatory B cells.

Generally considering, dendritic cells (DCs) are professional antigen-presenting cells (APCs) that process and present tumor antigens and initiate T cell responses to cancer cells. As studies reported, TEXs can impair DC proliferation and maturation as well as their functions. TEXs can interfere with monocytes differentiating into DC cells (Figure 1, (a)). In melanoma and colon cancer, TEXs could curb peripheral CD14^+^ monocytes differentiating into DC cells, while inducing them into myeloid-derived suppressor cells (MDSCs) [20]. Furthermore, TEXs could directly inhibit DCs' bioactivity and induce immune tolerance (Figure 1, (a)). TEXs force CD14^+^ monocyte to express HLA-DR at a low level [21]. In the presence of TEXs in culture medium, costimulatory molecule expression in human DCs were attenuated while inhibitory cytokines (e.g., TGF-*β* and PGE2) were elicited, with a dose-dependent suppression of T cell proliferation and antitumor cytotoxicity [22, 23]. What is more, TEXs of pancreatic cancers were indicated to downregulate the toll-like receptor 4 (TLR4) expression in DCs via miRNA-203, thus reducing downstream cytokines TNF-*α* and interleukin-12 (IL-12) [24].

## 3. Myeloid-Derived Suppressor Cells

Myeloid-derived suppressor cells (MDSC) is a well-known immune suppression factor that consists of immature myeloid cells including the precursors of DCs. Intravenous injected TEXs also showed marked accumulation of MDSCs in tumor, spleen, peripheral blood, and lung in vivo [24]. The accumulation of MDSCs could negatively affect the antigen processing and presentation and produce numerous immunosuppressive inhibitory factors, including NO and ROS, which cause TCRs nitration or T cell apoptosis [19]. Valenti et al. found that exosomes released by melanoma prohibit myeloid cells differentiating into DCs, while inducing them into TGF-*β*-secreting CD14^+^HLA-DR^−^ phenotype which was associated with suppressing T cell proliferation and cytotoxic functions [22]. CD14^+^HLA-DR^−^ MDSCs can also be found in patient's peripheral blood in many other malignancies, including hepatocellular carcinoma, bladder carcinoma, glioblastoma, and multiple myeloma [25–27]. An in vitro study indicated that TEX-driven MDSCs were capable to polarize normal monocytes to M2 phenotypes with higher expression of CD163, along with Th2 immune response and a tumor-promoting environment. Comparing to those in normal control, higher CD11b^+^CD14^+^HLA-DR^−^ TGF-*β* secreting cells could be found in the peripheral blood of stages II-III melanoma patients, but minor boost in stage IV patients [28–30]. This indicated that systematic MDSCs proliferation occurred in the early stage of neoplasm and melanoma released TEXs not only influenced the amount of MDSCs but also exerted impact on the differentiation of bone marrow to produce more immunosuppressive cell subsets [30]. Taylor and Gercel-Taylor confirmed that TEXs could activate the STAT1 and STAT3 pathways and increase antiapoptotic proteins Bcl-xL and Mcl-1 to prolong the survival of MDSCs [13]. TEXs could also boost NO releasing from MDSCs and enhance their suppressive activity in myeloma models. In TS/A mammary tumor murine model, TEXs injected into the bone marrow interacted with CD11b^+^ myeloid precursors, inducing IL-6 producing, Stat3 phosphorylation, and skewing bone marrow-derived cells (BMDCs) differentiation to MDSCs [31]. In breast cancer models, TEXs adopt TGF-*β* and IL-6 pathway to differentiate BMDCs towards MDSCs phenotype [32]. Chalmin et al. discovered that colon cancer TEXs with Hsp72-induced IL-6 toll-like receptor could accumulate MDSCs in mice and human beings [33–35]. Recent data also showed that MyD88 served as an important role in murine TEX-mediated MDSCs proliferation and contributed to lung metastasis through CCL2 in the C57BL/6J mice model [36]. Membrane-associated Hsp72 of TEXs can also trigger STAT3 activation in MDSCs through IL-6 via TLR2/MyD88 signal [33, 37]. But more functions of these TEX-related receptors needs to be further explored [33, 34, 38].

## 4. Macrophages

Macrophages are among the most abundant of innate immune cells that function as antitumor responses. In addition to phagocytes, macrophages can serve as cytokines and chemokines resource to recruit and induce other immune cells. Classically, macrophage can be activated by a range of environmental stimuli such as bacterial LPS and IFN-*γ*, can be transformed into M1-phenotype, and can enhance both innate and adaptive immunity. Studies have proved that M2 cytokine profile macrophages, also called tumor-associated macrophages, help enhance tumor metastasis and invasion. Cytokines such as CCL2, MIP2, IL-8, and IL-1R*α* that support tumor metastasis, angiogenesis, and protumor inflammation are upregulated, while the expression of antitumor cytokines such as TIMP-1, IFN-*γ*, IL-Ra, IL-13, and IL-16 are attenuated. As for the mechanism involved, proteins such as and Hsp72 and RNAs from TEXs have been shown to play a role through pattern recognition receptors (PRRs). Chow et al. demonstrated that palmitoylated proteins on TEXs can play as the ligand and bind to TLR2 on macrophages, stimulate NF-*κ*B signals in macrophages, and promote secretion of proinflammatory cytokines, such as IL-6, TNF-*α*, and CCL2 [39] (Figure 1, (b)). Fabbri et al. proved that TEX-derived miRNAs such as miR-21 and miR-29 served as the ligands of murines TLR7 and TLR8, leading to TLR-mediated NF-*κ*B activation in macrophage [40]. Another novel mechanism for the intercellular communication between cancer cells and tumor-associated macrophages is recently proposed by Menck et al. [41]. TEXs could induce the upregulation of Wnt 5*α* in macrophages and Wnt 5*α* could be delivered into tumor cells via macrophage-derived exosomes, thus leading to the activation of *β*-catenin-independent Wnt signaling in tumor cells and enhancing tumor invasion in breast cancer [41]. Recently, evidences proved that TEXs could also prolong tumor-associated macrophages survival in the inflammatory niche [29].

## 5. NK Cells

NK cells are the first-line defensive immune cells with cytotoxicity that directly kill tumor cells. TEXs contribute to immune escape via interfering the amount and function of NK cells. Whiteside showed that the percentage of NK cells in the spleen and lung decreased when treated with TEXs in mice models [42]. NK cells in tumor patients have a lower activity with less activation receptors such as NKp30, NKp46, NKG2C, and NKG2D [43, 44]. Among these receptors, NKG2D is the most critical one that binds to human MHC class I chain-related MICA and MICB to stimulate T cells' immune response. As the literature reported, TEXs can downregulate NKG2D expression, induce Smad phosphorylation, and reducing the cytolytic activity in NK cells [45] (Figure 1, (c)). In breast cancer and mesothelioma, tumor cells excreted NKG2D ligand containing TEXs to downregulate NKG2D expression, resulting in lower activity of NK cells [38, 46–48]. Apart from the NK receptors, other impaired signaling pathways contribute to less NK activation as well. In syngeneic BALB/c and nude mice models, TEXs released by TS/A or 4T.1 murine mammary tumor cell lines could intercept IL-2-mediated pathway to prevent NK cell activation and promote implanted tumor progression and metastasis [6]. In vitro and in vivo experiments demonstrate TEXs can also directly attenuate NK cell perforin and cyclin D3 expression, as well as the activation of JAK-3, furtherly inhibiting NK cell-mediated cytolysis [42]. In acute myeloid leukemia (AML), the serum soluble TGF-*β* also plays a role in TEX-associated NK cell dysfunction, which is consistent with the report that neutralizing antibodies against TGF-*β* could remove the TEX-induced inhibition [45].

## 6. Effector T Cells

It is believed that TEXs can both impair the activation of effector T cells and induce apoptosis of activated T cells in kinds of ways. Researchers found numerous malignant cells could release TEXs to induce T cell apoptosis, including nose pharynx cancer, pancreatic carcinoma, colon cancer, and gastric carcinoma [49–51]. Galectin-9, as the agonist of Tim-3, has been reported to be abundant in human nose pharynx cancer and served as a death-inducing receptor [52]. In Epstein-Barr virus-infected nose pharynx cancer, galectin-9 containing TEXs circulated to T cells and bind to Tim-3, thus inducing massive EBV-specific CD4^+^ lymphocyte apoptosis and inhibiting the function of Th1 cells [53]. Research findings suggest that TEXs could also express bioactive membrane-bound form of FasL and selectively induce T cell apoptosis via Fas/FasL interaction [6] (Figure 1, (d)). In vitro studies also showed that TEXs separated from malignant effusions such as ascites could also inhibit effector T cell activity through Fas/FasL interaction [49, 54, 55]. Besides, in ovarian carcinoma, TEXs utilize membrane-formed FasL to inhibit expression of CD3-*ζ* and further suppress the follow-up TCR signaling [56]. Andreola et al. discovered that melanoma TEXs not only expressed bioactive FasL but also specifically expressed CD63 and exosomal proteins, such as TRAIL, gp100, and MART-1 [57]. Both galectin-9 and Fas/FasL mechanisms are originally designed for T cell homeostasis control and self-limitation of immune response [58–61]. These research give us hints to understand that TEXs could circulate in the body and exert harmful effects on immune effector cells through some specific pathways, which might be the potential target of immunological therapy [49, 57, 62].

TEXs can impair the activation of T cell responses as well. TEXs could selectively inactivate CD8^+^ T cells by interfering with TCR- and IL-2R-mediated signaling [42] (Figure 1, (d)). In glioblastoma mice models, TEXs from glioblastoma GL26 cell line reduced the percentages of CD8^+^ T cells and inhibited the activation of CD8^+^ T cells, inducing decreased release of IFN-*γ* and granzyme B [45]. TCR signaling would be uncoupled by TEX-driven ROS burst, which would disrupt both CD4^+^ and CD8^+^ T cell signals and in turn downregulate T cell numbers [63].

Several studies have also demonstrated that exosomes can transport antigens from tumor cells to antigen-presenting dendritic cells [64–66]. Via MHC-I molecules, dendritic cells' prime cytotoxic T lymphocytes evoke an antitumor response and suppress tumor growth in vivo [65, 67]. Moreover, a potential direct presentation to T cells via HLA/peptide complex exosomal expression is also under investigation. Using these characteristics, modified TEXs could be designed as a tumor vaccine which would be discussed in Section 8.

Adenosine is another pathway that is related to T cell suppression. As one of the well-known immunosuppressive factors, adenosine has a role in T cell suppression by binding to its receptors (A1, A2A, A2B, and A3) [42, 68]. TEXs could increase the level of extracellular adenosine and thus decrease the local immunity. With the existence of both CD39 (ATP hydrolase) and CD73 (5′-nucleotidase) in cell surface, Treg cell could produce adenosine [69, 70]. TEXs could not only have activated CD39 and CD73 on the membrane surface but also directly deliver membrane-tethered CD73 to CD39^+^ cells, inducing the hydrolysis of ATP to adenosine and forming a T cell suppression environment [42, 71, 72].

## 7. Treg and Breg Cells

Most studies concentrated on the immunosuppressive effect of MDSC, lacking of research on regulatory T (Treg) cells and regulatory B (Breg) cells. TEXs could regulate other critical parts of the immune system, especially the impact on the immunosuppressive cells and cytokines. In addition to MDSCs, TEXs could enhance Treg and Breg proliferation and function [22, 23]. In vivo studies represented a crucial step for proving a true involvement of this pathway in immune suppression and tumor progression [6]. Myeloma patients' peripheral blood contains more CD4^+^CD25^+^FOXP3^+^ Treg cells than that of healthy donors, and high-concentrate TEXs could be found in the serum [27, 73]. Szajnik et al. reported TEXs separated from serum and ascites of cancer patients can phosphorylate Smad2/3 and Stat3, inducing CD4^+^CD25^−^ T cell transformed into CD4^+^CD25^+^Foxp3^+^ Treg cell [6, 73]. Clayton et al. found that TEXs could promote Treg cells and inhibit cytotoxicity cells via skewing IL-2 responsiveness [74]. TEXs with TGF-*β* upregulate Treg-related genes through TGF-*β*/Smad signaling activation and SAPK signaling devitalization in colorectal cancer [24]. Furthermore, TEXs can utilize the IL-10-dependent mechanism to promote the amount and function of Tregs and enhance the immunosuppression function [73, 75] (Figure 1, (e)). In vitro culture medium, TEXs could not only expand Tregs' amount and enhance Tregs' function but also help them be resistant to cell apoptosis [73]. Furthermore, Tregs showed a higher expression in FasL, IL-10, TGF-*β*, CTLA-4, and granzyme B and perforin in coculture with TEXs, as well as enhanced Smad2/3 and STAT3 phosphorylation [42, 73] (Figure 1, (e)). These TEX-mediated effects mainly rely on TGF-*β* and IL-10, while other molecules in TEXs such as miRNA-214-PTEN and EGFR might also be participated in the signaling pathway [73]. Antibodies designed for these cytokines can prevent TEXs from proliferating Treg cells [45].

Bregs are a unique subset of B cells which produce inhibitory cytokines and play suppressive roles in antitumor immune responses. High percentage and density of Bregs have been proved to be accumulated in the tissues and peripheral blood of invasive carcinoma of breast compared with that in patients with benign breast tumor or healthy women [76, 77]. Bregs expressing suppressive molecules such as IL-10, TGF-*β*, IL-35, IL-21, and PD-L1 can induce the generation of Tregs, upregulate MDSC function, and suppress CD4^+^ T cell-protective immune responses in both animal models and in vitro studies [77–81]. Recent research indicates that in murine splenocyte culture, exosomes from mycoplasma-infected tumor cells induce B cell-dependent IL-10 and suppress T cell activity [82]. Extracellular vesicles derived from esophageal cancer cells were also found to induce naive B cells to differentiate into TGF-*β*-producing Bregs which showed immune suppressor functions on CD8^+^ T cell proliferation [83]. Yang et al. discovered that mycoplasma-infected tumor cells could produce TEXs containing a component from mycoplasma, and these TEX-accumulated Breg cells in turn inhibit the activation of effector T cells [83] (Figure 1, (e)). These findings open a window to illustrate the mechanism of interaction between Breg cells and TEXs.

## 8. Immunotherapy Strategy

TEXs have been proved to play crucial roles in suppressing the immune system by attenuating the differentiation, proliferation, or functions of various immune cells. Thus, modulating the processes and reprogramming immune cells towards the opposite direction have great promises and might be effective. Relieving the suppression of TEXs on host immune system might be the key points for exosome-based immunotherapy.

**Figure 2:**
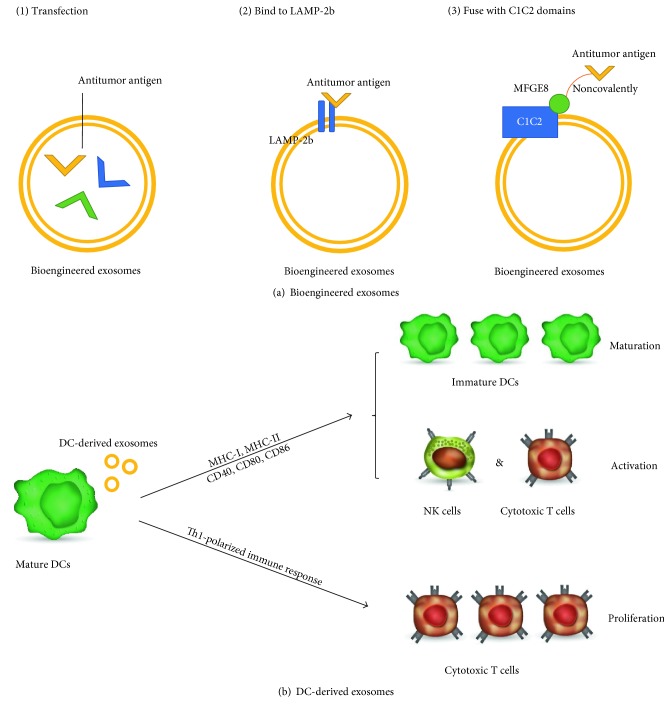
Exosomes serve as agents for immunotherapy strategy. (a) Three ways to create bioengineered exosomes: (1) Transfect exogenous antitumor antigen into exosomes directly; (2) bind the antitumor antigen onto exosome membrane surface protein LAMP-2b; (3) fuse antitumor antigen with lipid-binding C1C2 domains of the human lactadherin protein MFGE8 noncovalently. (b) Mature DCs produce DC-derived exosomes with MHC-I, MHC-II, and costimulatory molecules (CD40, CD80, and CD86) to induce immature DCs' maturation and active cytotoxic T cells and NK cells. DC-derived exosomes could also yield a Th1-polarized immune response to proliferate cytotoxic T cells.

Bioengineered exosomes mean load antitumor antigen into exosomes to produce a potent and antigen-specific immunostimulatory function. Now, there are 3 confirmed ways to import exogenous proteins into exosomes [84–88]. The first way is to use the transfection technique to load the exogenous proteins into exosomes directly [85, 86]. The second way is to bind onto the exosome membrane surface protein LAMP-2b [84]. The third way is to fuse the exogenous proteins with lipid-binding C1C2 domains of the human lactadherin protein (MFGE8) noncovalently [85, 87, 88] (Figure 2(a)). In vitro tests have been proved that importing immunostimulatory molecules into exosomes could yield immunogenicity of exosomes [89]. Immunostimulatory exosomes could be used as an immunogen for potent cancer vaccines in the future clinical use. But no successful data have been reported so far.

Alternatively, TEX can be used as a drug delivery system. Biological therapeutics, including short-interfering RNA and recombinant proteins, which are easy to degradation, have limitation in crossing the biological membranes and avoiding host immune responses [90, 91]. Exosomes as carriers for biological therapeutics could be served as a promising strategy to overcome these issues and to achieve efficient delivery to target cells [90]. However, the considerable complexity and the related high chance of off-target effects of these carriers are major barriers for clinical use [92]. Considering that not all components of exosomes are required for their proper function, artificial exosomes could be an alternative strategy [84, 93, 94]. But the necessary exosomal components required for the assembly of functional artificial exosomes remain to be identified [90, 94].

DC-derived exosomes work in totally different ways. This strategy focuses more on the host system and aims to reverse the tumor-induced immunosuppression. DC-derived exosomes are produced by mature DCs and can express MHC-I, MHC-II, and costimulatory molecules (CD40, CD80, and CD86) which could induce normal DCs' maturation and active cytotoxic T cells and natural killer (NK) cells [95–99] (Figure 2(b)). Besides, DC-derived exosomes could alter tumor-induced immunosuppression and activate innate and adaptive immune cells to induce antigen-specific responses against tumor cells [89, 99–101]. DC-derived exosomes could yield a Th1-polarized immune response to inhibit tumor antigen specifically in vivo, with IFN-*γ* accumulation and cytotoxic T cells proliferation [99–101] (Figure 2(b)). Following the promising preclinical animal studies, two phase I human clinical trials in melanoma and non-small-cell lung cancer using DC-derived exosome therapy have been completed. Only modest efficacy has been observed with no obvious toxicity [101, 102]. The authors suggest that these positive effects might be attributable to a small amount of NK cell activation [101, 102].

## 9. Conclusion

TEXs are rapidly emerging as a critical component which is designed to facilitate tumor immune escape and promote tumor growth. These TEXs could promote the differentiation of monocytes to MDSCs, educate macrophages into TAMs, inhibit NK cells activation, induce activated cytotoxic T cells apoptosis, and increase Tregs and Bregs, so as to suppress the host immune response. Due to these exosomal effects, they represent a central mediator of the tumor-supportive microenvironment. A large amount of research is emerging on the interaction between TEXs and host cells, which is still not fully clarified at present. Based on the known results, researchers have proposed some novel antitumor strategies including DC-derived exosomes and bioengineering of exosomes. In vitro and preclinical animal studies of these immunotherapy strategies have shown promising results, but there is still a long way to gain effective therapeutic effect in cancer patients. Actually, the responsible molecules including proteins and RNAs for TEXs-specific responses are still poorly understood, which leads to no systematic approach to generate TEXs with specified immune functions. Meanwhile, tumor microenvironment is essential for TEXs' contents and functions. Whether there is a batch of TEXs that could produce some immune stimulation effects is still doubtful. Additionally, the heterogeneous molecules within TEXs are involved in varieties of immunosuppressive or immune-stimulative signaling pathways, respectively. Thus, it is unclear whether changes in TEXs' contents or functions would occur when a specific molecule is loaded into or dissociated from TEXs, which directly influence the balance between tumor-promoting effects and antitumor effects of TEXs. Based on the above evidence and analysis, TEXs should not be thought as simple extracellular vesicles, but as bioactive vesicles with critical biological functions, which have great potential in cancer research and targeted therapy. Nevertheless, further researches are needed to illuminate the molecular mechanisms on TEX-specific effects and put forward more potent antitumor immunotherapy based on TEXs.
